# Air stacking: effects on pulmonary function in patients with spinal muscular atrophy and in patients with congenital muscular dystrophy*,**

**DOI:** 10.1590/S1806-37132014000500009

**Published:** 2014

**Authors:** Tanyse Bahia Carvalho Marques, Juliana de Carvalho Neves, Leslie Andrews Portes, João Marcos Salge, Edmar Zanoteli, Umbertina Conti Reed

**Affiliations:** Child Neurology Sector of the Department of Neurology, University of São Paulo School of Medicine Hospital das Clínicas, São Paulo, Brazil; Department of Neurology, University of São Paulo School of Medicine, São Paulo, Brazil; Department of Neurology, University of São Paulo School of Medicine, São Paulo, Brazil; Department of Neurology, University of São Paulo School of Medicine, São Paulo, Brazil; Department of Neurology, University of São Paulo School of Medicine, São Paulo, Brazil; Department of Neurology, University of São Paulo School of Medicine, São Paulo, Brazil

**Keywords:** Neuromuscular diseases, Cough, Respiratory function tests, Respiratory therapy

## Abstract

**OBJECTIVE:**

Respiratory complications are the main causes of morbidity and mortality in patients with neuromuscular disease (NMD). The objectives of this study were to determine the effects that routine daily home air-stacking maneuvers have on pulmonary function in patients with spinal muscular atrophy (SMA) and in patients with congenital muscular dystrophy (CMD), as well as to identify associations between spinal deformities and the effects of the maneuvers.

**METHODS:**

Eighteen NMD patients (ten with CMD and eight with SMA) were submitted to routine daily air-stacking maneuvers at home with manual resuscitators for four to six months, undergoing pulmonary function tests before and after that period. The pulmonary function tests included measurements of FVC; PEF; maximum insufflation capacity (MIC); and assisted and unassisted peak cough flow (APCF and UPCF, respectively) with insufflations.

**RESULTS:**

After the use of home air-stacking maneuvers, there were improvements in the APCF and UPCF. In the patients without scoliosis, there was also a significant increase in FVC. When comparing patients with and without scoliosis, the increases in APCF and UPCF were more pronounced in those without scoliosis.

**CONCLUSIONS:**

Routine daily air-stacking maneuvers with a manual resuscitator appear to increase UPCF and APCF in patients with NMD, especially in those without scoliosis.

## Introduction

Neuromuscular diseases (NMDs) are acquired or inherited conditions that affect parts of the neuromuscular system, such as skeletal muscles, peripheral motor nerves, neuromuscular junction, and motor neurons in the spinal cord.

Spinal muscular atrophy (SMA) is an autosomal recessive neurodegenerative disease of childhood characterized by degeneration and loss of lower motor neurons in the anterior horn cells of the spinal cord, causing progressive proximal weakness and atrophy of skeletal muscles.^(^ 1 ^,^ 2 ^)^ The cause of SMA is the homozygous mutation in the *SMN1* gene, and SMA is usually classified as one of four types, according to the age at onset and the maximum function attained^(^ 1 ^,^ 2 ^)^: type I (onset within the first six months of life and inability to sit without support); type II (onset after six months of age and inability to walk unaided); type III (onset after the two years of age and ability to walk at some point in life); and type IV (onset in adulthood).

Some of the most common inherited muscle diseases include progressive muscular dystrophies (e.g., Duchenne muscular dystrophy and limb-girdle muscular dystrophy) and congenital muscular dystrophy (CMD), the latter comprising a clinically and genetically heterogeneous group of muscular dystrophies that present within the first two years of life, characterized by hypotonia, muscle weakness, delayed motor development, and contractures.^(^ 3 ^)^ The most common forms of CMD involve deficiency of merosin, collagen VI, selenoprotein-N1, and lamin A/C, as well as of the glycosyltransferases involved in the glycosylation of alpha-dystroglycan protein.^(^ 3 ^)^

The main causes of morbidity and mortality in patients with NMD are the pulmonary complications that result from respiratory muscle weakness.^(4,5) ^Patients with NMD develop restrictive respiratory disorders caused by progressive weakening of the respiratory muscles and musculoskeletal deformities such as kyphoscoliosis.^(^ 6 ^,^ 7 ^)^ When NMD patients are unable to achieve an adequate PEF, they might also have impaired cough, as evidenced by a reduction in their peak cough flow (PCF), which results from inspiratory and expiratory muscle weakness.^(^ 8 ^)^ Ineffective cough (i.e., the inability to clear the airway by coughing) has been associated with PCF values of < 160 L/min.^(^ 9 ^)^ In addition, when the PCF is < 270 L/min, the risk of ineffective cough can increase during episodes of respiratory infection, impairing the removal of secretions and proper airway clearance, which can lead to respiratory failure.^(^ 5 ^,^ 9 ^,^ 10 ^)^

The maintenance of respiratory capacity is essential for the survival of patients with NMD, including those with minimal lung capacity. Therefore, it is relevant to investigate the effectiveness of air stacking, a technique that provides cough assistance, promoting improvement in respiratory capacity, as well as to helping reduce the risk of respiratory infections. The aim of this study was to determine the effects of routine daily air-stacking maneuvers on pulmonary function in patients with SMA or CMD. In addition, we aimed to identify associations between spine deformities and the effects of air stacking.

## Methods

In this longitudinal, uncontrolled study, we evaluated NMD patients treated at the NMD Outpatient Clinic of the University of São Paulo School of Medicine *Hospital das Clínicas* between February 2010 and August 2011. The diagnosis of CMD was confirmed by muscle biopsy, whereas that of SMA was confirmed by molecular testing. The inclusion criteria were showing an FVC < 90% of the predicted value, being over 6 years of age, never having been submitted to air-stacking maneuvers, and presenting with a level of cognition sufficient to understand the procedures that were to be performed. The exclusion criteria were having a concomitant lung disease, having a respiratory infection on the day of the assessment, having had a tracheostomy or being on noninvasive ventilation for more than 15 h/day, and using sedatives. Before and after the period of routine daily home air-stacking maneuvers, all patients underwent pulmonary function tests and were classified as underweight, normal, overweight, or obese, according to their body mass index (BMI).

The study was approved by the Research Ethics Committee of the Hospital das Clínicas (Protocol no. 0087/09). All participating patients or their legal guardians gave written informed consent.

### Evaluation of pulmonary function

All pulmonary function tests were performed by the same respiratory therapist, who used a pneumotachograph and a spirometer (Spirolab II; Medical International Research, Rome, Italy) to measure FVC, PEF, PCF, and assisted PCF (APCF, assisted by air stacking to the maximum pulmonary volume with a manual resuscitator), in randomized order and in accordance with the Brazilian Thoracic Association Guidelines for Pulmonary Function Tests.^(^ 11 ^)^ All measurements were performed with the subject in a seated position, with the head in a neutral position. The subject wore a face mask that covered the nose and mouth, connected to the pneumotachograph and the spirometer. Patients were asked to inhale as deeply as possible, then perform a forced expiratory maneuver down to residual volume and sustain that for at least three seconds. Three measurements were taken, and the best of the three was considered in the analyses, as long as the difference between any two of the three measurements was no more than 0.15 L. For each subject, we obtained at least three measurements with three acceptable curves and two reproducible curves per measurement. From those curves, we computed the values of FVC and PEF. Predicted values were calculated with the reference equations for spirometry in Brazil, which vary according to gender and age bracket (6-13 years; 14-19 years; and ≥ 20 years).^(^ 12 ^-^ 16 ^)^ Unassisted PCF (UPCF) was measured with the subject in a seated position, wearing a face mask that covered the nose and mouth; the subject was asked to perform a maximal inspiration followed by a cough. A maximum of six coughs or attempts to cough were allowed, and the best value obtained was considered in the analyses, as long as the difference between any two of the three measurements was no more than 20 L/min.

### Determination of maximum insufflation capacity

Using a manual resuscitator, we determined the maximum insufflation capacity (MIC) after air stacking from the volume delivered to the patient via a face mask connected to the pneumotachograph and the spirometer. We performed three consecutive manual insufflations while requesting that the patient take a deep breath and hold it (with a closed glottis), stacking breaths to achieve the MIC. During each insufflation, the examiner used a thumb to block the valve of the air outlet of the T-piece of the resuscitator. At the end of each insufflation, the thumb was removed and the patient exhaled the maximal volume of held air into the system, which measured the MIC. We determined the APCF by following that same protocol, except for the last step, in which, rather than exhaling, the patient was asked to cough and the PCF was recorded. The highest of the PCFs recorded in a maximum of six coughs or attempts to cough was considered in the analyses.

### Air stacking protocol

All patients received a manual resuscitator with an attached face mask of a size proportional to the face of the patient, in order to perform the routine daily air-stacking maneuvers at home for four to six months, the duration depending on the time since the last evaluation. At the NMD Outpatient Clinic, patients and caregivers were trained in the air-stacking technique and were instructed to perform the maneuvers with the patient in a sitting position, the caregiver positioned behind the patient, and the mask connected to the manual resuscitator. The prescribed daily regimen was 10 series of three to four consecutive manual insufflations, the patient taking deep, sustained breaths (held with a closed glottis) during each series. The patients were instructed to hold the total volume (after the final insufflation) for eight seconds and then exhale. The patients and caregivers were instructed to divide the exercises into three sessions per day, to be performed in the morning, afternoon, and evening.

### Statistical analysis

All data were analyzed with GraphPad Prism software, version 5.0 (GraphPad Software, Inc., San Diego, CA, USA). The anthropometric characteristics were expressed as mean ± standard deviation. All numeric variables of the study were subjected to the D'Agostino-Pearson normality test. The pre- and post-training anthropometric and spirometric data were compared with Student's t-tests. Comparisons regarding different parameters were analyzed with unpaired Student's t-tests, as were comparisons between the patients with and without scoliosis. We also employed Pearson's correlation coefficients to investigate associations between UPCF and FVC; between UPCF and MIC; between PEF and MIC; and between the MIC-FVC difference (ΔMIC-FVC) and the APCF-UPCF difference (ΔAPCF-UPCF). In all cases, the level of significance was p < 0.05.

## Results

We recruited 22 patients with NMD. Three patients were lost to follow-up, and another was excluded because of respiratory complications that led to the need for tracheostomy. Therefore, the final sample comprised 18 patients (10 females and 8 males; 7-23 years of age), all of whom completed the respiratory evaluations. Ten patients were diagnosed with CMD, 4 were diagnosed with SMA type II, and 4 were diagnosed with SMA type III. 

The mean ages and anthropometric characteristics of the patients, before and after the training, are shown, by diagnosis, in Table 1. There was a statistically significant post-training increase in the mean height of the patients. The mean BMI of the patients did not change. Six patients who were under 20 years of age presented alterations in BMI-4 were underweight, 1 was obese, and 1 was overweight. Two patients who were over 20 years of age were underweight. Nine patients were found to have structural scoliosis. None of the patients had undergone any kind of respiratory therapy or had been on noninvasive ventilation prior to enrollment in the study.

**Table 1:** Ages and anthropometric data for 18 patients with neuromuscular diseases, before and after the training (4-6 months of routine daily home air-stacking maneuvers).a

Characteristic	Pre-training	Post-training
Age (years)	15.39 ± 5.50	15.72 ± 5.37
Height (cm)	151.7 ± 14.82	152.6 ± 14.78*
Weight (Kg)	40.50 ± 10.26	40.78 ± 10.20
BMI	17.65 ± 4.16	17.60 ± 4.23

BMI: body mass index

a Data are presented as mean ± SD

* p < 0.05 vs. pre-training (paired Student's t-test).

### Pulmonary function variables

In terms of the pulmonary function variables, there were no statistical differences between the SMA and CMD groups, before or after the training (Table 2). In the sample as a whole, the mean values for FVC, MIC, and PEF did not differ significantly between the pre- and post-training periods, although there were significant post-training increases in the mean UPCF and mean APCF (Table 3). The increases in UPCF and APCF were less pronounced in the patients with scoliosis than in those without. In addition, there was a significant post-training increase in the mean FVC of the patients without scoliosis, whereas no such increase was observed in the patients with scoliosis (Table 4).

**Table 2:** Pulmonary function variables in 18 patients with neuromuscular diseases, before and after the training (4-6 months of routine daily home air-stacking maneuvers), by diagnosis.

Variable	Pre-training		Post-training	
	SMA	CMD	SMA	CMD
	(n = 8)	(n = 10)	(n = 8)	(n = 10)
FVC (L)	1.818 ± 0.652	1.758 ± 0.580*	1.835 ± 0.625	1.824 ± 0.668*
MIC (L)	2.044 ± 0.732	2.047 ± 0.585*	2.084 ± 0.676	2.036 ± 0.706*
UPCF (L/min)	237.30 ± 85.48	274.10 ± 84.10*	261.90 ± 75.19	290.80 ± 102.80*
APCF (L/min)	248.40 ± 73.86	292.20 ± 313.3*	283.10 ± 85.16	313.30 ± 110.10*

SMA: spinal muscular atrophy; CMD: congenital muscular dystrophy; MIC: maximum insufflation capacity; UPCF: unassisted peak cough flow; and APCF: assisted peak cough flow

a Data are presented as mean ± SD

* p > 0.05 vs. SMA (unpaired Student's t-test).

**Table 3:** Pulmonary function variables in 18 patients with neuromuscular diseases, before and after the training (4-6 months of routine daily home air-stacking maneuvers).

Variable	Pre-training	Post-training
FVC (L)	1.784 ± 0.595	1.829 ± 0.631
MIC (L)	2.046 ± 0.634	2.057 ± 0.673
PEF (L/min)	175.80 ± 89.04	191.3 ± 96.90
UPCF (L/min)	257.80 ± 84.31	277.90 ± 90.24*
APCF (L/min)	272.70 ± 82.92	299.80 ± 98.19*

MIC: maximum insufflation capacity; UPCF: unassisted peak cough flow; and APCF: assisted peak cough flow. aData are presented as mean ± SD

* p < 0.0001 vs. pre-training (paired Student's t-test).

**Table 4:** Pulmonary function variables in 18 patients with neuromuscular diseases, with and without scoliosis, before and after the training (4-6 months of routine daily home air-stacking maneuvers).a

Variable	Pre-training		Post-training	
	Without scoliosis	With scoliosis	Without scoliosis	With scoliosis
	(n = 9)	(n = 9)	(n = 9)	(n = 9)
FVC (L)	2.10 ± 0.332	1.469 ± 0.646	2.191 ± 0.315*	1.467 ± 0.672
MIC (L)	2.357 ± 0.323	1.734 ± 0.729	2.409 ± 0.278	1.706 ± 0.778
UPCF (L/min)	295.50 ± 54.99	220 ± 94.19	315.60 ± 51.50^†^	240.30 ± 107.10
APCF (L/min)	299.10 ± 57.56	246.30 ± 98.65	334.80 ± 52.16^‡^	264.90 ± 122.5

MIC: maximum insufflation capacity; UPCF: unassisted peak cough flow; and APCF: assisted peak cough flow

a Data are presented as mean ± SD

* p < 0.05 vs. pre-training (paired Student's t-test)

† p < 0.01 vs. pre-training (paired Student's t-test)

‡ p < 0.0001 vs. pre-training (paired Student's t-test).

**Figure 1:**
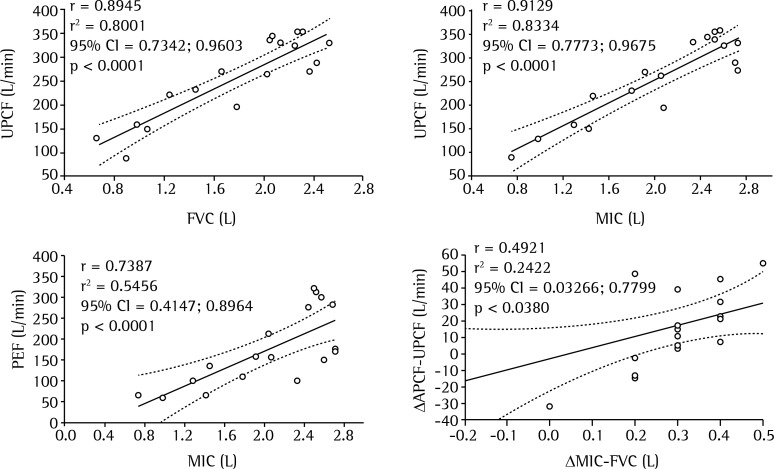
Correlation analysis of pre-training data. UPCF: unassisted peak cough flow; MIC: maximum insufflation capacity; APCF: assisted peak cough flow; ?APCF-UPCF: difference between APCF and UPCF; and ?MIC-FVC: difference between MIC and FVC.

As depicted in Figure 1, a comparison analysis of the pre-training data revealed a correlation between UPCF and FVC; between UPCF and MIC; between PEF and MIC; and between ΔAPCF-UPCF and ΔMIC-FVC (Figure 1).

## Discussion

While it was not possible to verify adherence to the regimen of routine daily home air-stacking maneuvers, we observed increases in UPCF and APCF among NMD patients for whom such exercises were prescribed. We also observed an increase in FVC among the patients without scoliosis. In addition, the increases in UPCF and APCF were much less pronounced in the patients with scoliosis than in those without.

Patients with NMD show a decrease in muscle strength and consequently lose the ability to take spontaneous deep breaths. Therefore, these patients initially develop microatelectasis and eventually develop permanent pulmonary restriction.^(^ 17 ^)^ The ability to cough efficiently is correlated with the clearance of airway secretions. Patients who are unable to perform air-stacking maneuvers effectively can take deep breaths with the aid of a volumetric ventilator at a pressure of 40 cmH_2_O. Thus, through regular maximal insufflations, it is possible to increase the MIC and dynamic lung compliance.^(^ 10 ^)^ The availability of such devices facilitates the care of patients with NMD, even at the early stages of respiratory muscle impairment, because the assisted maneuvers or the PCF produced with the aid of these devices are ideal for the mechanical reproduction of a cough. However, their high cost limits the ability of patients to acquire such instruments.

Assisted cough techniques have been shown to be critical in preventing episodes of respiratory failure that lead to hospitalization and the need for tracheostomy in patients with NMD.^(^ 4 ^)^ However, only a few studies have investigated the effects of air-stacking maneuvers on the pulmonary function of patients with NMD,^(^ 10 ^,^ 18 ^,^ 19 ^)^ and there have been no reports of previous studies employing this methodology in patients with CMD. 

In the present study, the PCF was higher after the training. The steady expansion of the lungs with daily deep breaths not only improves inspiratory capacity, but also allows for increased lung distension and more efficient gas exchange; this offers better ventilation to the lungs and increases the volume of voice and PCF, leading to a reduction in microatelectasis and an improvement in lung compliance.^(^ 8 ^,^ 10 ^,^ 20 ^-^ 22 ^)^ Although the spirometry maneuvers employed in order to assess PEF and PCF are similar, each requires a different combination of respiratory muscle groups. The closing of the glottis increases the transpulmonary pressure created by coughing. However, the effectiveness of a cough depends on the PCF, which is greater if the airways are narrowed during coughing; this is more effective in removing secretions than is simply performing a forced expiratory maneuver.^(^ 23 ^)^

Patients with NMD also show a decrease in FVC-a marker of the development and progression of the disease-due to progressive weakening of the respiratory muscles and to spinal deformities, which leads to decreased volumes and reduced lung expansion.^(^ 6 ^,^ 20 ^,^ 24 ^)^ The benefits of air-stacking maneuvers in patients with diminished FVC were demonstrated by Bach et al.^(^ 20 ^)^ During the short period of training evaluated in the present study, no improvement of FVC was noted for the sample as a whole. However, there was a significant post-training improvement in FVC in the subgroup of patients without scoliosis. This finding suggests that chest deformities not only impair lung function^(^ 25 ^,^ 26 ^)^ but also have a negative effect on the response to air-stacking exercises. 

In the present study, we found that PCF correlated with FVC and MIC. Effective PCF and PEF both require high lung volumes, which explains their good correlation with FVC and MIC. The correlation between UPCF and MIC, as well as the correlation between ΔAPCF-UPCF and ΔMIC-FVC, is explained by their dependence on bulbar innervated muscles and on the integrity of the glottic function. The PCF is also dependent on the permeability of the hypopharynx being maintained by innervation of the bulbar muscles of the hypopharyngeal musculature.^(^ 27 ^-^ 29 ^)^ In addition, glottic function is the most important factor in protecting the airways and maintaining cough efficacy in patients with NMD.^(^ 10 ^)^

The present study has some significant limitations, such as the heterogeneity and small size of the patient sample, the difficulty that younger patients have in performing air-stacking maneuvers, the unsupervised nature of the home application of the maneuvers, the lack of comparisons between APCF and abdominal thrust, and the lack of a control group (due to ethical issues). Nevertheless, we can conclude that home air-stacking maneuvers should be prescribed for patients with NMD, in order to increase their PCF.
