# Supplementary material for: Air stacking: effects on pulmonary function in patients with spinal muscular atrophy and in patients with congenital muscular dystrophy
Source: J Bras Pneumol. 2014 Sep-Oct;40(5):528–34. doi: 10.1590/S1806-37132014000500009 (PMC4263334; doi:10.1590/S1806-37132014000500009)
Supplement: Translation

# Translation: Efeitos do treinamento de empilhamento de ar na função pulmonar de pacientes com amiotrofia espinhal e distrofia muscular congênita*

**Authors:** Tanyse Bahia Carvalho Marques, Juliana de Carvalho Neves, Leslie Andrews Portes, João Marcos Salge, Edmar Zanoteli, Umbertina Conti Reed

**Affiliations:** Disciplina de Neurologia Infantil, Departamento de Neurologia, Hospital das Clínicas, Faculdade de Medicina, Universidade de São Paulo, São Paulo (SP) Brasil; Departamento de Neurologia, Faculdade de Medicina, Universidade de São Paulo, São Paulo (SP) Brasil; Departamento de Neurologia, Faculdade de Medicina, Universidade de São Paulo, São Paulo (SP) Brasil; Departamento de Neurologia, Faculdade de Medicina, Universidade de São Paulo, São Paulo (SP) Brasil; Departamento de Neurologia, Faculdade de Medicina, Universidade de São Paulo, São Paulo (SP) Brasil; Departamento de Neurologia, Faculdade de Medicina, Universidade de São Paulo, São Paulo (SP) Brasil

**Keywords:** Doenças neuromusculares, Tosse, Testes de função respiratória, Terapia respiratória

## Abstract

**OBJETIVO:**

As complicações respiratórias são as principais causas de morbidade e mortalidade em pacientes com doenças neuromusculares (DNM). Os objetivos deste estudo foram determinar os efeitos que o treinamento diário domiciliar com manobras de empilhamento de ar tem na função respiratória de pacientes com amiotrofia espinhal (AE) e distrofia muscular congênita (DMC), e identificar possíveis associações entre deformidades na coluna vertebral e os efeitos das manobras.

**MÉTODOS:**

Dezoito pacientes com DNM (dez com DMC e oito com AE) foram submetidos a treinamento diário domiciliar de empilhamento de ar com ressuscitador manual por um período de quatro a seis meses e submetidos a testes de função pulmonar antes e após tal período. Os testes de função pulmonar incluíram medidas de CVF, PFE, a capacidade de insuflação máxima (CIM) e a medida do pico de fluxo de tosse não assistido e assistido (PFTNA e PFTASS, respectivamente).

**RESULTADOS:**

Após o uso das manobras de empilhamento de ar no domicílio, houve uma melhora significativa na PFTNA e PFTASS. Nos pacientes sem escoliose, houve também um aumento significativo na CVF. No grupo de pacientes sem escoliose, o ganho na PFTNA e PFTASS foi superior ao do grupo com escoliose.

**CONCLUSÕES:**

A utilização rotineira diária de manobras de empilhamento de ar com ressuscitador manual parece melhorar a PFTNA e PFTASS em pacientes com DNM, especialmente naqueles sem escoliose.

## Introdução

As doenças neuromusculares (DNM) são doenças adquiridas ou hereditárias que afetam partes do sistema neuromuscular, tais como músculos esqueléticos, nervos motores periféricos, junção neuromuscular, e neurônios motores da medula espinhal. 

A amiotrofia espinhal (AE) é uma doença neurodegenerativa autossômica recessiva da infância; caracteriza-se pela degeneração e perda de neurônios motores inferiores nas células do corno anterior da medula espinhal e causa fraqueza proximal progressiva e atrofia muscular esquelética.^(^ 1 ^,^ 2 ^)^ A AE é causada pela mutação em homozigose no gene *SMN1* e é geralmente classificada em quatro tipos, de acordo com a idade de início e a função máxima alcançada^(^ 1 ^,^ 2 ^)^: tipo I (início nos primeiros seis meses de vida e incapacidade de se sentar sem apoio); tipo II (início depois de seis meses de idade e incapacidade de andar sem ajuda); tipo III (início depois de dois anos de idade e capacidade de andar em algum momento da vida) e tipo IV (início na idade adulta). 

Dentre as doenças musculares hereditárias mais comuns estão distrofias musculares progressivas (como, por exemplo, a distrofia muscular de Duchenne e a distrofia muscular de cinturas) e a distrofia muscular congênita (DMC), que compreende um grupo clinicamente e geneticamente heterogêneo de distrofias musculares que surgem nos dois primeiros anos de vida e que se caracteriza por hipotonia, fraqueza muscular, desenvolvimento motor atrasado e contraturas.^(^ 3 ^)^ As formas mais comuns de DMC envolvem deficiência de merosina, colágeno VI, selenoproteína-N1 e lamina A/C, bem como das glicosiltransferases envolvidas na glicosilação da proteína alfa-distroglicana.^(^ 3 ^)^

As principais causas de morbidade e mortalidade em pacientes com DNM são as complicações pulmonares resultantes da fraqueza muscular respiratória.^(^ 4 ^,^ 5 ^)^ Pacientes com DNM apresentam doenças respiratórias restritivas causadas pelo enfraquecimento progressivo dos músculos respiratórios e por deformidades musculoesqueléticas tais como a cifoescoliose.^(^ 6 ^,^ 7 ^)^ Quando pacientes com DNM são incapazes de alcançar um PFE adequado, é possível que apresentem também tosse prejudicada, evidenciada pela redução do pico de fluxo da tosse (PFT), que resulta da fraqueza muscular inspiratória e expiratória.^(^ 8 ^)^ Estabeleceu-se uma relação entre a tosse ineficaz (isto é, a incapacidade de desobstruir a via aérea por meio da tosse) e valores de PFT < 160 L/min.^(^ 9 ^)^ Além disso, quando o PFT é < 270 L/min, o risco de tosse ineficaz pode aumentar durante episódios de infecção respiratória, prejudicando a remoção de secreções e a desobstrução adequada das vias aéreas, o que pode levar a insuficiência respiratória.^(^ 5 ^,^ 9 ^,^ 10 ^)^

A manutenção da capacidade respiratória é essencial para a sobrevivência de pacientes com DNM, inclusive daqueles com capacidade pulmonar mínima. Portanto, é relevante investigar a eficácia do empilhamento de ar, uma técnica de assistência à tosse que melhora a capacidade respiratória e ajuda a reduzir o risco de infecções respiratórias. O objetivo deste estudo foi determinar os efeitos de manobras diárias de empilhamento de ar na função pulmonar de pacientes com AE ou DMC. Além disso, buscamos identificar associações entre deformidades da coluna vertebral e os efeitos do empilhamento de ar. 

## Métodos

Neste estudo longitudinal não controlado, avaliamos pacientes com DNM atendidos no Ambulatório de DNM do Hospital das Clínicas da Faculdade de Medicina da Universidade de São Paulo entre fevereiro de 2010 e agosto de 2011. O diagnóstico de DMC foi confirmado por meio de biópsia muscular, ao passo que o de AE foi confirmado por meio de teste molecular. Os critérios de inclusão foram CVF < 90% do previsto, idade > 6 anos, nunca ter realizado manobras de empilhamento de ar e apresentar um nível de cognição suficiente para compreender os procedimentos que seriam realizados. Os critérios de exclusão foram doença pulmonar concomitante, infecção respiratória no dia da avaliação, traqueostomia ou ventilação não invasiva durante mais de 15 h/dia e uso de sedativos. Antes e depois do período de manobras de empilhamento de ar realizadas diariamente em casa, todos os pacientes foram submetidos a testes de função pulmonar e foram classificados em abaixo do peso, normais, acima do peso ou obesos, de acordo com seu índice de massa corporal (IMC). 

O estudo foi aprovado pelo Comitê de Ética em Pesquisa do Hospital das Clínicas (Protocolo no. 0087/09). Todos os participantes ou responsáveis assinaram um termo de consentimento livre e esclarecido. 

### Avaliação da função pulmonar

Todos os testes de função pulmonar foram realizados pelo mesmo fisioterapeuta respiratório, que usou um pneumotacógrafo e um espirômetro (Spirolab II; Medical International Research, Roma, Itália) para medir a CVF, o PFE, o PFT e o PFT assistido (PFTASS, assistido por empilhamento de ar até o volume pulmonar máximo com um ressuscitador manual), em ordem aleatória e de acordo com as Diretrizes para Testes de Função Pulmonar da Sociedade Brasileira de Pneumologia e Tisiologia.^(^ 11 ^)^ Todas as medições foram realizadas com os pacientes sentados, com a cabeça em posição neutra. Os pacientes usavam uma máscara facial que cobria o nariz e a boca e se conectava ao pneumotacógrafo e ao espirômetro. Os pacientes eram instruídos a inspirar o mais profundamente possível e, em seguida, executar uma manobra expiratória forçada até o volume residual e mantê-la durante pelo menos três segundos. Foram realizadas três medições, e a melhor das três foi usada nas análises, contanto que a diferença entre quaisquer duas das três medições não fosse maior que 0.1.5 L. Para cada paciente, foram obtidas pelo menos três medidas com três curvas aceitáveis e duas curvas reprodutíveis por medição. A partir dessas curvas, foram computados os valores de CVF e PFE. Os valores previstos foram calculados por meio das equações de referência para a espirometria no Brasil, que variam de acordo com o gênero e a faixa etária (6-13 anos; 14-19 anos e ≥ 20 anos).^(^ 12 ^-^ 16 ^)^ O PFT não assistido (PFTNA) foi medido com os pacientes sentados e usando uma máscara facial que cobria o nariz e a boca; os pacientes eram instruídos a realizar uma inspiração máxima seguida de uma tosse. Foram permitidas no máximo seis tosses ou tentativas de tosse, e o melhor valor obtido foi usado nas análises, contanto que a diferença entre quaisquer duas das três medidas não fosse maior que 20 L/min. 

### Determinação da capacidade de insuflação máxima

Usando um ressuscitador manual, determinamos a capacidade de insuflação máxima (CIM) após o empilhamento de ar a partir do volume fornecido ao paciente por meio de uma máscara facial conectada ao pneumotacógrafo e ao espirômetro. Foram realizadas três insuflações manuais consecutivas enquanto o paciente inspirava profundamente e prendia a respiração (com a glote fechada), empilhando o ar para alcançar a CIM. Durante cada insuflação, o examinador usava um polegar para bloquear a válvula da saída de ar do tubo T do ressuscitador. No fim de cada insuflação, o polegar era removido e o paciente expirava o volume máximo de ar, até então preso, para dentro do sistema, que media a CIM. Determinamos o PFTASS seguindo esse mesmo protocolo, à exceção do último passo, no qual, em vez de expirar, o paciente era orientado a tossir e o PFT era registrado. O maior dos PFT registrados em no máximo seis tosses ou tentativas de tosse foi usado nas análises. 

### Protocolo de empilhamento de ar

Todos os pacientes receberam um ressuscitador manual conectado a uma máscara facial de tamanho proporcional à face do paciente, a fim de realizar as manobras diárias de empilhamento de ar em casa durante quatro a seis meses; a duração dependia do tempo decorrido desde a última avaliação. No Ambulatório de DNM, os pacientes e cuidadores receberam treinamento para realizar a técnica de empilhamento de ar e foram instruídos a realizar as manobras com o paciente sentado e o cuidador posicionado atrás do paciente, e a máscara conectada ao ressuscitador manual. O regime diário prescrito foi de 10 séries de três a quatro insuflações manuais consecutivas, com o paciente realizando inspirações profundas e mantendo a respiração presa com a glote fechada durante cada série. Os pacientes foram instruídos a prender o volume total (após a insuflação final) durante oito segundos e então expirar. Os pacientes e cuidadores foram instruídos a dividir os exercícios em três sessões por dia (manhã, tarde e noite). 

### Análise estatística

Todos os dados foram analisados com o programa GraphPad Prism, versão 5.0 (GraphPad Software, Inc., San Diego, CA, EUA). As características antropométricas foram expressas em média ± desvio-padrão. Todas as variáveis numéricas do estudo foram submetidas ao teste de normalidade de D'Agostino-Pearson. Os dados antropométricos e espirométricos pré e pós-treinamento foram comparados por meio de testes-t de Student. Comparações de diferentes parâmetros foram analisadas por meio de testes-t de Student não pareados, assim como o foram as comparações dos pacientes com e sem escoliose. Foram também empregados coeficientes de correlação de Pearson para investigar associações entre PFTNA e CVF; entre PFTNA e CIM; entre PFE e CIM e entre a diferença entre CIM e CVF (ΔCIM-CVF) e a diferença entre PFTASS e PFTNA (ΔPFTASS-PFTNA). Em todos os casos, o nível de significância foi de p < 0,05. 

## Resultados

Foram recrutados 22 pacientes com DNM. Três pacientes foram perdidos, e outro foi excluído em virtude de complicações respiratórias que levaram à necessidade de traqueostomia. Portanto, a amostra final consistiu em 18 pacientes (10 mulheres e 8 homens; 7-23 anos de idade), todos os quais completaram as avaliações respiratórias. Dez pacientes receberam diagnóstico de DMC, 4 receberam diagnóstico de AE tipo II e 4 receberam diagnóstico de AE tipo III. 

As médias de idade e as características antropométricas dos pacientes, antes e depois do treinamento, são apresentadas, por diagnóstico, na Tabela 1. Depois do treinamento, houve um aumento significativo da média de altura dos pacientes. A média do IMC dos pacientes não se alterou. Seis pacientes com menos de 20 anos de idade apresentaram alterações no IMC - 4 estavam abaixo do peso, 1 era obeso e um estava acima do peso. Dois pacientes com mais de 20 anos de idade estavam abaixo do peso. Nove pacientes apresentavam escoliose estrutural. Nenhum dos pacientes havia sido submetido a qualquer tipo de fisioterapia respiratória ou recebera ventilação não invasiva antes da inclusão no estudo. 

**Tabela 1:** Idades e dados antropométricos de 18 pacientes com doenças neuromusculares, antes e depois do treinamento (4-6 meses de manobras de empilhamento de ar realizadas diariamente em casa).a

Característica	Pré-treinamento	Pós-treinamento
Idade (anos)	15,39 ± 5,50	15,72 ± 5,37
Estatura (cm)	151,7 ± 14,82	152,6 ± 14,78*
Peso (kg)	40,50 ± 10,26	40,78 ± 10,20
IMC	17,65 ± 4,16	17,60 ± 4,23

IMC: índice de massa corporal. aDados apresentados na forma de média ± dp

* p < 0,05 vs. pré-treinamento (teste t de Student pareado).

### Variáveis de função pulmonar

No tocante às variáveis de função pulmonar, não houve diferenças estatísticas entre os grupos AE e DMC, antes ou depois do treinamento (Tabela 2). Na amostra como um todo, não houve diferenças significativas entre as médias de CVF, CIM e PFE pré e pós- treinamento, embora tenha havido aumentos significativos das médias de PFTNA e PFTASS depois do treinamento (Tabela 3). Os aumentos de PFTNA e PFTASS foram menos pronunciados nos pacientes com escoliose do que naqueles sem o desvio. Além disso, depois da intervenção, houve um aumento significativo da média da CVF dos pacientes sem escoliose, o qual não foi observado nos pacientes com escoliose (Tabela 4). 

**Tabela 2:** Variáveis de função pulmonar em 18 pacientes com doenças neuromusculares, antes e depois do treinamento (4-6 meses de manobras de empilhamento de ar realizadas diariamente em casa), por diagnóstico.

Variável	Pré-treinamento		Pós-treinamento	
	AE	DMC	AE	DMC
	(n = 8)	(n = 10)	(n = 8)	(n = 10)
CVF (L)	1,818 ± 0,652	1,758 ± 0,580*	1,835 ± 0,625	1,824 ± 0,668*
CIM (L)	2,044 ± 0,732	2,047 ± 0,585*	2,084 ± 0,676	2,036 ± 0,706*
PFTNA (L/min)	237,30 ± 85,48	274,10 ± 84,10*	261,90 ± 75,19	290,80 ± 102,80*
PFTASS (L/min)	248,40 ± 73,86	292,20 ± 313,3*	283,10 ± 85,16	313,30 ± 110,10*

AE: amiotrofia espinhal; DMC: distrofia muscular congênita; CIM: capacidade de insuflação máxima; PFTNA: pico de fluxo da tosse não assistido; e PFTASS: pico de fluxo da tosse assistido

a Dados apresentados na forma de média ± dp

* p > 0,05 vs. AE (teste t de Student não pareado).

**Tabela 3:** Variáveis de função pulmonar em 18 pacientes com doenças neuromusculares, antes e depois do treinamento (4-6 meses de manobras de empilhamento de ar realizadas diariamente em casa).

Variável	Pré-treinamento	Pós-treinamento
CVF (L)	1,784 ± 0,595	1,829 ± 0,631
CIM (L)	2,046 ± 0,634	2,057 ± 0,673
PFE (L/min)	175,80 ± 89,04	191,3 ± 96,90
PFTNA (L/min)	257,80 ± 84,31	277,90 ± 90,24*
PFTASS (L/min)	272,70 ± 82,92	299,80 ± 98,19*

CIM: capacidade de insuflação máxima; PFTNA: pico de fluxo da tosse não assistido; e PFTASS: pico de fluxo da tosse assistido

a Dados apresentados na forma de média ± dp

* p < 0,0001 vs. pré-treinamento (teste t de Student pareado).

**Tabela 4:** Variáveis de função pulmonar em 18 pacientes com doenças neuromusculares, com e sem escoliose, antes e depois do treinamento (4-6 meses de manobras de empilhamento de ar realizadas diariamente em casa).a

Variável	Pré-treinamento		Pós-treinamento	
	Sem escoliose	Com escoliose	Sem escoliose	Com escoliose
	(n = 9)	(n = 9)	(n = 9)	(n = 9)
CVF (L)	2,10 ± 0,332	1,469 ± 0,646	2,191 ± 0,315*	1,467 ± 0,672
CIM (L)	2,357 ± 0,323	1,734 ± 0,729	2,409 ± 0,278	1,706 ± 0,778
PFTNA (L/min)	295,50 ± 54,99	220 ± 94,19	315,60 ± 51,50^†^	240,30 ± 107,10
PFTASS (L/min)	299,10 ± 57,56	246,30 ± 98,65	334,80 ± 52,16^‡^	264,90 ± 122,5

CIM: capacidade de insuflação máxima; PFTNA: pico de fluxo da tosse não assistido; e PFTASS: pico de fluxo da tosse assistido. aDados apresentados na forma de média ± dp

* p < 0,05 vs. pré-treinamento (teste t de Student pareado)

† p < 0,01 vs. pré-treinamento (teste t de Student pareado)

‡ p < 0,0001 vs. pré-treinamento (teste t de Student pareado).

Como mostra a Figura 1, uma análise comparativa dos dados pré-treinamento revelou correlações entre PFTNA e CVF; entre PFTNA e CIM; entre PFE e CIM; e entre ΔPFTASS- PFTNA e ΔCIM-CVF (Figura 1). 

**Figura 1:** Análise de correlação de dados no pré-treinamento. PFTNA: pico de fluxo da tosse não assistido; CIM: capacidade de insuflação máxima; PFTASS: pico de fluxo da tosse assistido; ?PFTASS-PFTNA: diferença entre PFTASS e PFTNA; e ?CIM-CVF: diferença entre CIM e CVF.

## Discussão

Embora não tenha sido possível verificar a adesão ao regime de manobras domiciliares diárias de empilhamento de ar, observamos aumentos de PFTNA e PFTASS em pacientes com DNM para os quais esses exercícios foram prescritos. Observamos também um aumento da CVF nos pacientes sem escoliose. Além disso, os aumentos de PFTNA e PFTASS foram muito menos pronunciados nos pacientes com escoliose do que naqueles sem o desvio. 

Pacientes com DNM apresentam força muscular reduzida e, consequentemente, perdem a capacidade de respirar fundo espontaneamente. Portanto, esses pacientes inicialmente apresentam microatelectasias e, finalmente, restrição pulmonar permanente.^(^ 17 ^)^ A capacidade de tossir eficientemente correlaciona-se com a depuração de secreções das vias aéreas. Os pacientes que não são capazes de realizar manobras de empilhamento de ar de maneira eficaz podem respirar profundamente com o auxílio de um ventilador volumétrico a 40 cmH_2_O de pressão. Assim, por meio de insuflações máximas regulares, é possível aumentar a CIM e a complacência pulmonar dinâmica.^(^ 10 ^)^ A disponibilidade desses dispositivos facilita a assistência a pacientes com DNM, mesmo nos estágios iniciais de comprometimento da musculatura respiratória, pois as manobras assistidas ou o PFT produzido com a ajuda desses dispositivos são ideais para a reprodução mecânica da tosse. No entanto, o elevado custo limita a capacidade dos pacientes de adquirir tais instrumentos. 

Demonstrou-se que técnicas de tosse assistida são fundamentais para prevenir episódios de insuficiência respiratória que levam a hospitalização e à necessidade de traqueostomia em pacientes com DNM.^(^ 4 ^)^ No entanto, apenas alguns estudos investigaram os efeitos das manobras de empilhamento de ar na função pulmonar de pacientes com DNM,^(^ 10 ^,^ 18 ^,^ 19 ^)^ e não há relatos de estudos anteriores que tenham empregado essa metodologia em pacientes com DMC. 

No presente estudo, o PFT foi maior após o treinamento. A constante expansão dos pulmões com respirações profundas diárias não só melhora a capacidade inspiratória, mas também permite maior distensão pulmonar e troca gasosa mais eficiente; isso propicia melhor ventilação para os pulmões e aumenta o volume da voz e o PFT, levando a uma redução das microatelectasias e a uma melhora na complacência pulmonar. ^(^ 8 ^,^ 10 ^,^ 20 ^-^ 22 ^)^ Embora as manobras espirométricas empregadas a fim de avaliar o PFE e o PFT sejam semelhantes, cada uma delas requer uma combinação diferente de grupos de músculos respiratórios. O fechamento da glote aumenta a pressão transpulmonar criada pela tosse. No entanto, a eficácia da tosse depende do PFT, que é maior se as vias aéreas se estreitam durante a tosse; isso é mais eficaz na remoção de secreções do que simplesmente realizar uma manobra expiratória forçada.^(^ 23 ^)^

Pacientes com DNM apresentam também uma diminuição da CVF - um marcador do surgimento e da progressão da doença - em virtude do enfraquecimento progressivo dos músculos respiratórios e de deformidades da coluna vertebral, o que leva a volumes reduzidos e expansão pulmonar reduzida.^(^ 6 ^,^ 20 ^,^ 24 ^)^ Os benefícios das manobras de empilhamento de ar em pacientes com CVF reduzida foram demonstrados por Bach et al.^(^ 20 ^)^ Durante o curto período de treinamento avaliado no presente estudo, não se observou nenhuma melhora na CVF da amostra como um todo. No entanto, após o treinamento, houve uma melhora significativa na CVF no subgrupo de pacientes sem escoliose. Esse achado sugere que deformidades torácicas não só prejudicam a função pulmonar,^(^ 25 ^,^ 26 ^)^ mas também têm um efeito negativo na resposta a exercícios de empilhamento de ar. 

No presente estudo, o PFT correlacionou-se com a CVF e a CIM. Para que tanto o PFT como o PFE sejam eficazes, são necessários altos volumes pulmonares, o que explica sua boa correlação com a CVF e a CIM. Tanto a correlação entre PFTNA e CIM como a correlação entre ΔPFTASS-PFTNA e ΔCIM-CVF são explicadas por sua dependência de músculos bulbares inervados e da integridade da função glótica. O PFT também depende da manutenção da permeabilidade da hipofaringe por meio da inervação dos músculos bulbares da musculatura da hipofaringe.^(^ 27 ^-^ 29 ^)^ Além disso, a função glótica é o fator mais importante na proteção das vias aéreas e manutenção da eficácia da tosse em pacientes com DNM.^(^ 10 ^)^

O presente estudo tem algumas limitações significativas, tais como a heterogeneidade e o pequeno tamanho da amostra de pacientes, a dificuldade que os pacientes mais jovens têm em realizar manobras de empilhamento de ar, a natureza não supervisionada da aplicação domiciliar das manobras, a ausência de comparações entre PFTASS e compressão abdominal e a ausência de um grupo controle (por questões éticas). Não obstante, podemos concluir que manobras domiciliares de empilhamento de ar devem ser prescritas para pacientes com DNM, a fim de aumentar seu PFT.
